# Ventilation and air-conditioning systems in dental clinics and COVID-19: How much do we know?

**DOI:** 10.4317/jced.58119

**Published:** 2021-07-01

**Authors:** Sonia P. Plaza-Ruiz, Diana M. Barbosa-Liz, Andrés A. Agudelo-Suárez

**Affiliations:** 1 Orthodontic Posgraduate Program. Faculty of Dentistry, Fundación Universitaria CIEO-UniCIEO. Bogotá, Colombia; 2 Orthodontic Posgraduate Program. Faculty of Dentistry. University of Antioquia, Medellin, Colombia; 3 Faculty of Dentistry. University of Antioquia, Medellin, Colombia

## Abstract

**Background:**

This study evaluated the association between knowledge and management of ventilation and air-conditioning systems (VAC) to avoid the spread of the SARS-CoV-2 virus in health facilities by dentists and demographic variables.

**Material and Methods:**

A cross-sectional digital media survey was administered to dentists as part of global research. The core questionnaire was used including four additional questions on VAC (Q1: knowledge, Q2: work settings, Q3: temperature, and Q4: maintenance). A descriptive analysis was conducted for sociodemographic and VAC variables, and bivariate analysis was carried out using different tests.

**Results:**

5370 dentists answered the survey (median age of 45 years; 72.22% women). About half of the respondents said that they knew about the guidelines issued for the management of air conditioners (AC) during the pandemic, and 16.77% have made modifications to their VAC systems during this period. The most frequent AC temperature range used in the dentists’ offices during the pandemic was 18°C to 20°C. As age increased, self-reported knowledge about VAC guidelines expanded. Remote and rural regions were perceived to have less knowledge of the guidelines.

**Conclusions:**

Although perceptions of knowledge about VAC systems during the COVID-19 pandemic was high, the temperature in dental offices was colder than that recommended. Greater disclosure of VAC management practices and adherence to VAC management guidelines are required.

** Key words:**Air conditioning, dentistry, coronavirus.

## Introduction

The 2019 coronavirus disease (COVID-19) pandemic, caused by infection from severe acute respiratory syndrome coronavirus 2 (SARS-CoV-2), is seriously affecting the world. COVID-19 transmission via respiratory droplets has been demonstrated (1), but airborne transmission has also been discussed, with many researchers supporting the hypothesis of aerosol-driven infection by SARS-CoV-2 as an important transmission pattern and mechanism by which the disease spreads (2,3). The World Health Organization (WHO) recognized the possibility of airborne transmission indoors, especially in crowded and poorly ventilated places (3).

Since the occurrence of the SARS epidemic in 2003, the impact of heating, ventilation and air-conditioning (HVAC) systems in hospital environments has been widely studied (4-6). Researchers agreed that the ventilation and filtration provided by these systems can reduce the airborne concentration of viruses, and thus, the risk of transmission through the air. Inappropriate usage, however, may contribute to virus propagation. Ventilation enables air from the outdoors to flow into a building or room by natural or mechanical means (7). Diverse studies indicated that three elements of ventilation affecting airborne transmission are ventilation rate (VR), flow direction, and airflow pattern (2,8,9). One of the first cases of an outbreak associated with air conditioning-driven transmission of SARS-CoV-2 was in a restaurant in Guangzhou, China and involved three family-based clusters (10).

Dental healthcare workers (DHWs) are at a high risk of COVID-19 contagion because they are in close contact with patients, which presents the possibility of transmission via droplets when patients cough, sneeze, or talk. Virus carriers can also spread the disease via aerosolized saliva or aerosol-generating procedures. Despite the important role of HVAC systems in the transmission of SARS-CoV-2 in dental environments, few studies have focused on it. Among them is the research of Zemouri *et al*. (11), who constructed a mathematical model of the transmission of coronaviruses and other viral diseases in dental clinics. The authors concluded that improving indoor air quality through ventilation is the most important factor to take into account when controlling the transmission of SARS-CoV-2.

The WHO and other organizations devoted to the control of HVAC systems, such as the American Society of Heating, Refrigeration and Air Conditioning Engineers (ASHRAE), the Federation of European Heating, Ventilation, and Air Conditioning Associations (REHVA), the Colombian chapter of ASHRAE, and the Colombian Association of Air Conditioners (ACAIRE), among others, has been issuing different guidelines on the use of HVAC systems in health environments to avoid the spread of viruses and disease transmission (12). Since the declaration of the COVID-19 pandemic, some of these guidelines have been adequate to prevent its spread (12). However, it is not clear if dentists know them and/or apply them in their offices. Having access to this information could help to consolidate public health policies and their dissemination, reinforcement, and adherence by DHWs in their practices could reinforce other measures to prevent the spread of different viruses in dental settings. The aim of the present study was to evaluate the association between knowledge and management of ventilation and air-conditioning systems (VAC) to avoid the spread of the SARS-CoV-2 virus in health facilities by dentists and demographic variables.

## Material and Methods

Study setting and population: Following the protocol and core global questionnaire described by Campus *et al*. (13), a cross-sectional anonymous survey was administered online to dentists (general or specialists) working in both national health systems and private or public clinics in Colombia. A Colombian research team for COVID-19, of which the researchers in the present study are members, incorporated into the core questionnaire four additional questions regarding knowledge (Q1), work settings (Q2), temperature (Q3), and maintenance of VAC systems (Q4) to avoid the spread of the SARS-CoV-2 virus in health facilities (13).

-Sample size calculation: The sample was collected by convenience sampling from the initial population of 31,872 dentists registered to work in Colombia in 2019. The required sample size [calculated from the data obtained in the pilot study, using the OpenEpi software (Open-source epidemiologic statistics for public health version 3.01, AG Dean, KM Sullivan, MM Soe, Atlanta, GA, USA)] of 2,510 subjects was obtained. A 99.9% confidence level was considered and an estimated prevalence of VAC knowledge of 20% was given to establish an estimate within ±3% of this value. The inclusion criteria included dentists who worked in Colombia. Surveys with errors in the information record were excluded.

-Questionnaire development and evaluation: Three researchers formed the panel of experts who incorporated the four additional questions into the VAC systems questionnaire and validated it. Thirty randomly selected (simple random sampling) dentists who work in Colombia voluntarily participated in the pilot test. Amongst them, 10 were instructed to assess face validity (Does the test ‘look like’ a measure of the construct of interest?) and semantic comprehension, whereas the remaining 20 were asked to assess test–retest reliability (application of the instrument repeatedly at two time points, separated by 4-7 days).

-Survey administration: Google Forms was used to create the questionnaire, and the survey sent required cutting and pasting a web link into emails, social media posts, or websites to be distributed by dental associations Facebook groups, WhatsApp messages, email messages, and institutional invitations sent to different dental schools in Colombia. The country’s dental unions, such as the Colombian Dental Federation, and all specialist associations were convened. The survey was administered along with a promotional video that was launched over the aforementioned digital media. The Google Forms settings were adjusted in order to disallow unanswered or incomplete questions and modified to not show the link again after the response was sent. To address the issue of information bias, the questionnaire was forwarded by mail only to dentists who had not previously opened it. Additionally, in the introduction and final part of the survey, a warning was sent to not fill it out again if it had been filled out previously. No incentives were offered to the participants.

-Survey period: The survey was run from June 19 to July 24, 2020.

-Ethical approval: The survey was granted approval by the Ethics Committee of UniCIEO University (Number 101, minute 62). Following the Campus *et al*. (13) protocol, all the respondents of the survey were asked to answer an informed consent question on the first page of the questionnaire. An answer of ‘yes’ automatically signified agreement to participate. The data were organized on an Excel spreadsheet.

-Variables: The dependent variables were the four items (knowledge, work settings, temperature, and maintenance) mentioned in the VAC systems questionnaire, and the independent variables were sex, geographic region, and age. Age was treated as a continuous variable, but for purposes of statistical analysis, it was categorized as follows: young adult (22–35 years old), adult (36–45 years old), mature adult (46–59 years old) and older adult (≥60 years old).

-Statistical analyses: The completely anonymized data were analyzed by one of the researchers and checked for consistency and quality. Statistical analyses were performed using STATA 16 (StataCorp, College Station, Texas). A descriptive analysis was directed towards the sociodemographic and VAC variables, and a bivariate analysis was carried out using a chi-square test or Fisher’s exact test. In all the tests, a *P*<0.05 was considered indicative of significance, but this was adjusted in the Bonferroni correction for multiple testing to *P*<0.004 as the significance cut-off point.

## Results

Test-retest reliability was high with a kappa coefficient from 0.84 to 0.96 (CI:0.61;0.98). In total, 5,375 completed surveys were obtained; however, five surveys were discarded due to errors in the information record, leaving 5,370 viable surveys. With a total of 31,872 dentists registered to work in the Colombian territory, the corresponding response rate of the survey was of 16.84%.

The descriptive statistics are presented in Table 1, which shows that participants had a median age of 45 years (range: 22-82 years), and the majority were women (72.22%).

**Table 1:**
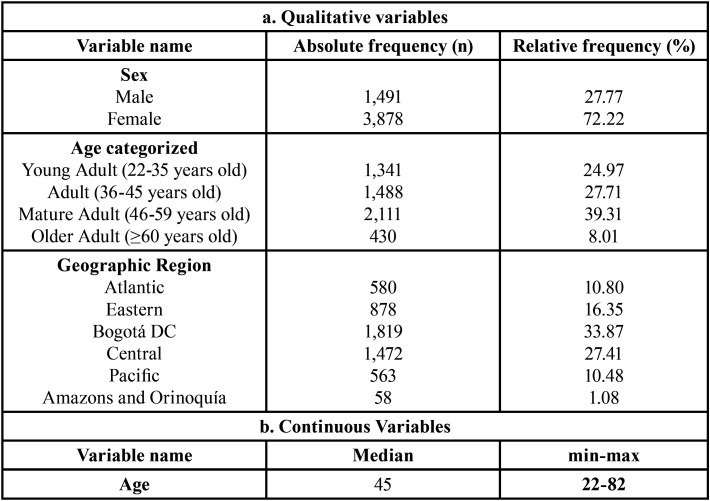
Descriptive characteristics of the sample.

With regard to questions about the knowledge and management of VAC systems to avoid the spread of the SARS-CoV-2 virus in their clinical practices, the researchers excluded the response ‘not applicable (N/A)’ as the participating dentists were limited to those with dental practices (clinicians). About half of the dentists said that they knew about the guidelines for the management of air conditioners during the COVID-19 pandemic. Of the clinicians who had air conditioning (AC) in their offices (37.81%), half operated the systems with windows open, whereas the other half did so with windows closed. Only 16.77% have made modifications to their AC systems during the COVID-19 pandemic. Among the clinicians, 44.19% did not use AC and worked with windows open (Table 2).

**Table 2:**
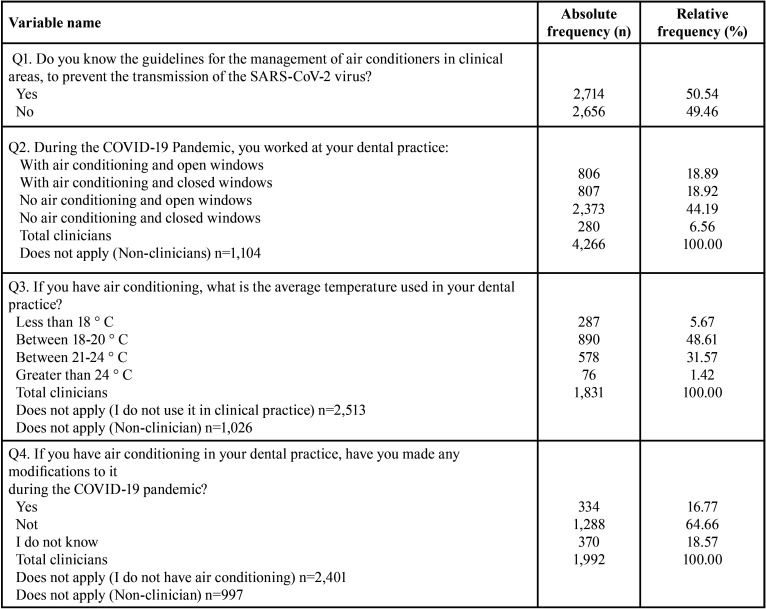
Dentists knowledge and management of VAC systems.

Tables  Table 3,  Table 3 cont. and  Table 4,  Table 4 cont. show the results of the association between sociodemographic variables (sex, categorized age, and region) and the questionnaire items. We found evidence of association (*P*<0.04) for most of the studied variables, except with respect to Q1 and Q3 and sex. A higher percentage of women (49.56%) than men (46.88%) reported awareness of the guidelines for the management of air conditioners in clinical areas as a means of preventing SARS-CoV-2 transmission (Q1). As regards Q3 (‘What is the average temperature used in your dental practice?’), a higher percentage of men (36.04%) used the recommended temperature range for AC in health facilities (21°C–24°C) compared with women (29.32%), but these associations were not significant. Of the dentists who used AC, a higher percentage of men worked with windows open (21.88% vs. 17.67%), and among those who did not use AC, more women worked in the aforementioned conditions (57.45% Vs 51.25%) (Q4). More women (21.21%) than men (13.34%) did not know whether they had made any modifications to their AC systems during the COVID-19 pandemic (Q4).

**Table 3:**
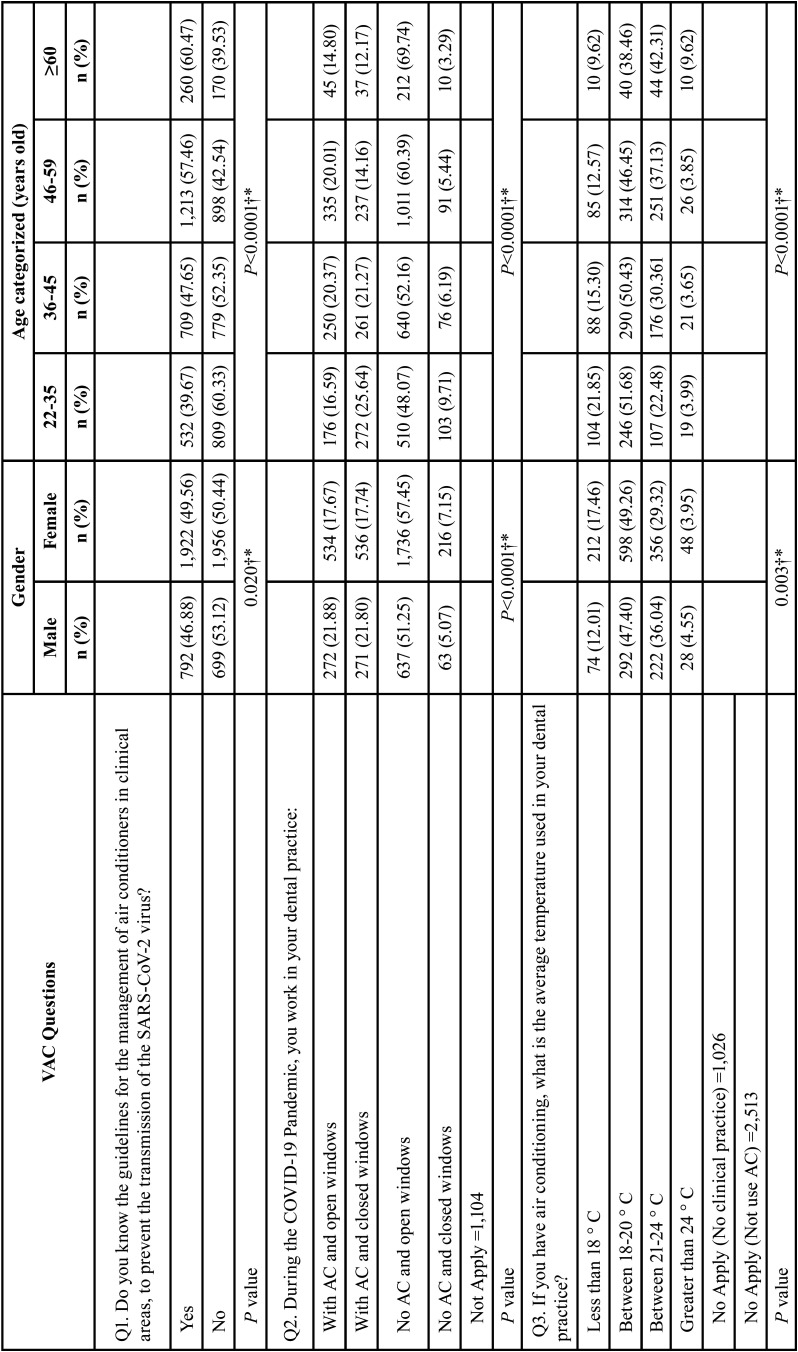
Bivariate analysis between VAC questions and gender and age.

**Table 3 cont.**
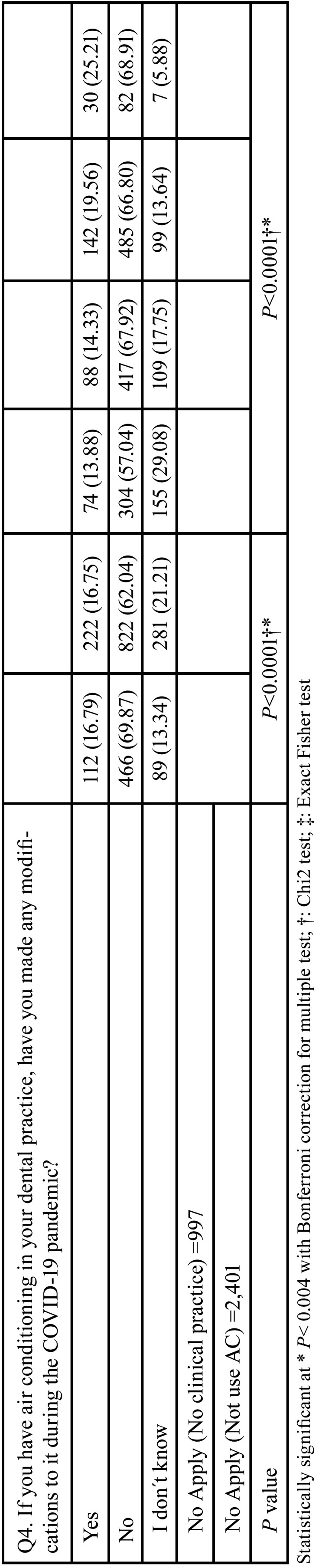
Bivariate analysis between VAC questions and gender and age.

**Table 4:**
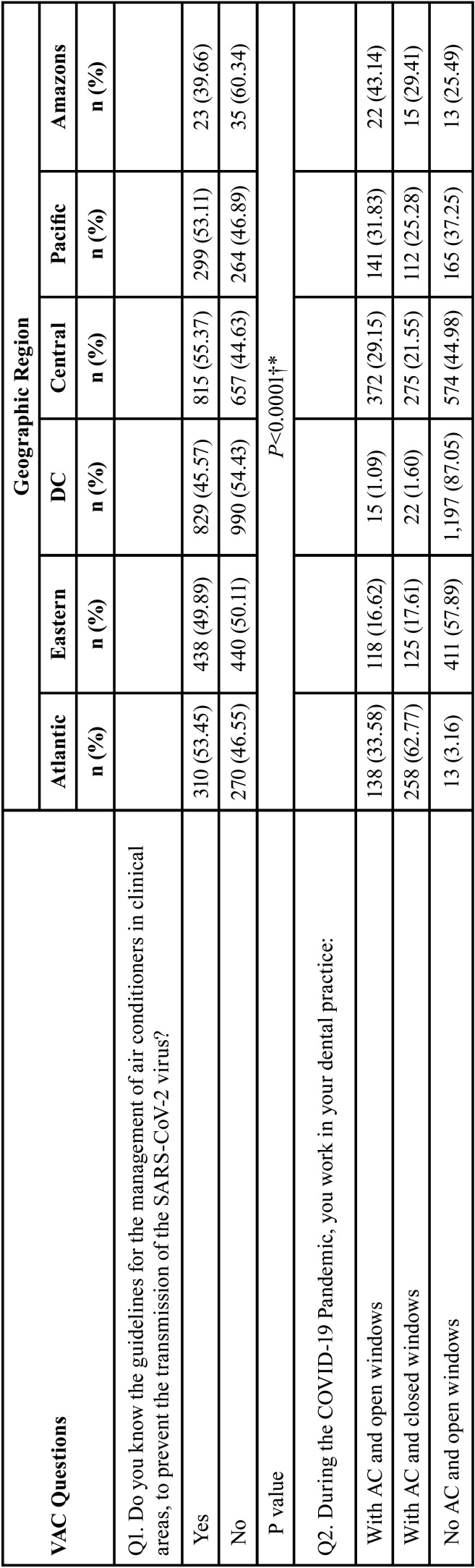
Bivariate analysis between VAC questions and geographic regions.

**Table 4 cont.**
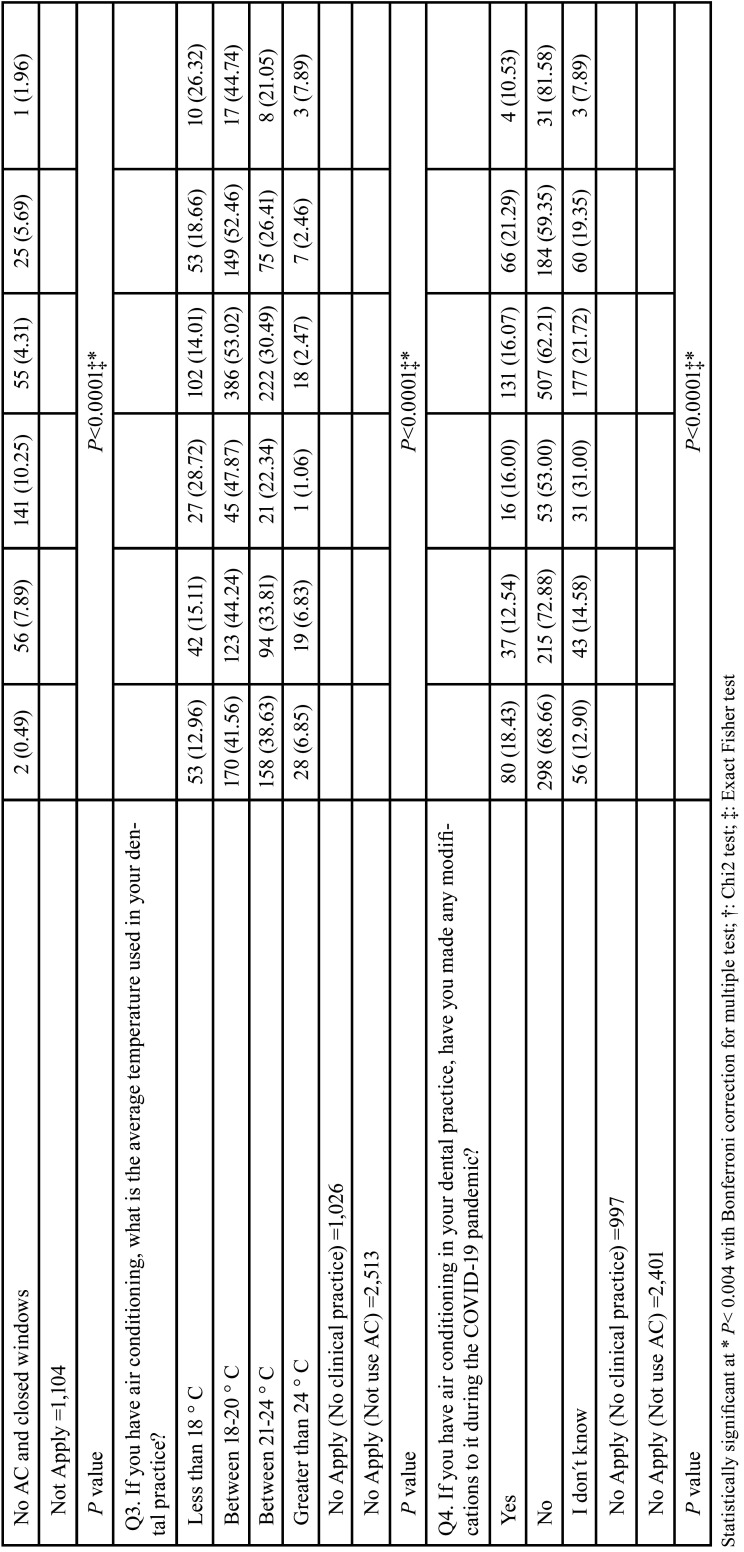
Bivariate analysis between VAC questions and geographic regions.

We found that as age increased, the perceived knowledge about the guidelines for the management of air conditioners in clinical areas increased. In the dental clinics with no AC and closed windows, an inverse relationship between knowledge and age was found. The older age group who did not use AC tended to work with windows open. A higher percentage of the clinicians aged between 36 and 59 years who worked with AC and windows open, as well as the older groups, tended to work in conformity with the recommended temperature range to avoid the spread of the SARS-CoV-2 virus in health facilities.

The remote regions of Colombia (Amazons and Orinoquía) were perceived to have less knowledge of the recommended guidelines for AC in health facilities, whereas the urban regions, as central areas of the country, were regarded as having greater knowledge. More frequent use of AC was observed in the Atlantic region (96.35%), whereas less frequent use was registered in Bogotá (2.69%). Most of the clinicians in Bogotá worked with windows open (87.05%). In all regions, the most frequently applied AC temperatures fell between 18°C and 20°C.

## Discussion

This research describes the knowledge and management of ventilation and air-conditioning systems (VAC) by dentists in Colombia during the COVID-19 pandemic to avoid the spread of the SARS-CoV-2 virus in health facilities and its association with sex, age, and geographic region. Our sample consisted of a very high percentage of women (72.22%), which may be related to the higher proportion of registered female dentists in Colombia. Regarding age, the representation observed in all age groups in the sample is similar to that reported by the special registry of health providers (REPS) in Colombia. Therefore, the sample of the present study could be considered representative of the dentists working in Colombia.

We found that 50.54% of the participants perceived themselves to have good knowledge of the recommended guidelines, but only 31.57% followed the recommended mean AC temperatures (21°C–24°C), with these practitioners preferring cooler temperatures. Given that dentistry is considered a high-risk profession because of the easy spread of viral agents in the air during dental procedures and the persistence of biological agents in operating rooms (14), new recommendations for biosecurity protocols and the use of personal protective equipment (PPE) have been proposed (15,16). Among these recommendations is the use of particulate respirators, eye protection, gowns, and gloves during dental procedures (17). Although studies have shown that using N95 masks does not result in significant clinical changes in body temperature (14), wearing all necessary PPE at the same time could cause an increase in body temperature. This can explain the preference for cooler temperatures among the clinicians in our study. According to the Amoatey *et al*. study (9), the lack of indoor building ventilation and the low indoor air temperature due to the excessive use of air conditioning systems increase infection by SARS-CoV-2.

The current results showed that among the surveyed dentists who have been using AC (n=1,613) at work during the COVID-19 pandemic, almost 50% (n=806) worked with windows open, and more of these individuals were men. Out of those who did not use AC (n=2,642), a higher percentage (89.8%) worked with windows open, especially women. These findings suggest that dentists in Colombia have more effectively managed natural ventilation than mechanical ventilation during the pandemic. However, scientific evidence indicates that the use of AC and the opening of windows are not obligatory, but a suggestion meant to increase ventilation (18). Another important consideration is energy consumption when AC is running while windows are left open (19). This reduces operating efficiency and undermines efforts to maintain mean temperatures in rooms. However, Sihwan Lee (18) explored the energy loss that occurs through doors in commercial stores and found that it does not result in increased electric power consumption.

With respect to the maintenance of or modifications to AC systems during the COVID-19 pandemic, only 16.77% of the dentists who had AC have implemented these measures, and more women than men did not know whether these measures were carried out in their clinics. In general, knowledge of technical specifications for VAC systems among clinicians is rare because of the technical language and numerous settings associated with such equipment. This could be a limitation for clinicians’ adherence to the guidelines.

We also found that age was associated with the knowledge and practice of AC and COVID-19 regulations. With more perceived knowledge about the recommended guidelines on the management of air conditioners in clinical areas, the majority of the older age group conformed to the recommended temperatures, used AC while their windows were open, and implemented AC maintenance. One aspect that can explain the greater self-reported knowledge of the recommended guidelines of VAC systems management during the COVID-19 pandemic among the older dentists in the current work is the nature of COVID-19, with the disease exerting a more serious impact on older individuals (20).

Colombia is located slightly north of the Equator and has different climatic areas and relative humidity and very little variation in a given year. The country’s geographical make-up factored somewhat into the results of the study because VAC systems are commonly used in some areas of the country, such as the Atlantic and Pacific regions where temperatures fall between 27°C and 34°C. In other regions, such as Bogotá, VAC systems are not necessary as these locations have a mean temperature falling between 9°C and 18°C. This could explain the greater use of AC in the Atlantic region (96.35%) and the less frequent use in Bogotá (2.69%) as well as the most frequently adopted AC temperatures of 18°C to 24°C in all the regions of the country.

As for perceived knowledge about the recommended guidelines, we found that rural regions, such as the Amazon and Orinoquía, had less perceived knowledge of the guidelines than that observed in urban/central areas. Rural areas have limited access to information given restrictions on Wi-Fi services and reduced Internet connection speeds. A valuable strategy is to ensure that hard-copy documents with recommended instructions and government regulations are physically sent to remote regions with connectivity problems. The findings also indicated that in cold regions, such as Bogotá and some cites in the central region, where AC systems are not needed, dentists preferred to work with windows open. Natural ventilation is very important in controlling virus proliferation in health care settings (4,6,11).

Knowledge and application of aspects such as modifications to VAC airflow patterns, ventilation, humidity, temperatures, and filter types, among others, are critical for clinicians because they can contribute to the control of virus proliferation, specifically in building service operation (8). This, despite being an aspect unrelated to dental practice, requires consideration by clinicians, because performing dental procedures in a well-ventilated location may help prevent the spread of infections (2).

One limitation of our study was the potential of sampling bias, because the sample was chosen by convenience, as well as the response and non-response bias. Another limitation could be that the survey was collected during a concise period of time, when the COVID-19 outbreak in the country was at a specific stage, and this could have affected the responses of those surveyed since some regions had higher response rates than others. These limitations of the study could affect the generalizability of the results.

Our study gave particular attention to temperature range, rather than ventilation rates. This could be a limitation as ventilation rates play an important role in spread. However, dentists may not be familiar with this information. It could be suggested to dentists that they seek advice from professional engineers to ensure the proper settings of the VAC systems in their clinics.

## Conclusions

Although the perception of knowledge about the management of VAC systems to avoid the spread of SARS-CoV-2 virus in health facilities was high, it is necessary for clinicians to follow recommended guidelines and maintain and monitor all VAC systems in their offices and clinics.
